# Masonic Signet and Journal

**Published:** 1859-07

**Authors:** 


### MASONIC SIGNET AND JOURNAL.

We have received several numbers of the Masonic Signet and Journal, and desire to direct the attention of all those, whether Masons or otherwise, who would desire to know more of this ancient and honorable fraternity, to this Journal, as the source from which to derive a great variety of valuable and interesting information in regard to the objects, usages and workings of Masonry.

The Journal is edited by Samuel Lawrence, of Marietta, Ga., (to whom all communications, for its pages, and exchanges, should be addressed,) and W. T. C. Campbell, of Atlanta, Ga. All letters on business and subscription should be addressed to Lawrence & Campbell, Atlanta, Ga., where it is published, monthly, at $2 per annum, in advance.

